# Thiamethoxam-Induced Acute Methemoglobinemia in a Child

**DOI:** 10.7759/cureus.87194

**Published:** 2025-07-02

**Authors:** Sania Shahid, Tuqa A Abdulsalam, Mitra A Ghafoor Zeyaei, Nada Ben Mechlia

**Affiliations:** 1 Pediatric Emergency Medicine, Al Jalila Children's Speciality Hospital, Dubai, ARE

**Keywords:** accidental toxic ingestion, insecticide toxicity, methemoglobinemia, methylene blue therapy, methylene blue treatment, neonicotinoid insecticide, pediatric poisoning, thiamethoxam gel, thiamethoxam toxicity

## Abstract

Methemoglobinemia is a rare and life-threatening condition in which the presence of methemoglobin (MetHb) impairs oxygen delivery to tissues. While hematologic toxicity is well-documented with agents like nitrates, benzocaine, and certain drugs, it is rarely associated with neonicotinoid insecticides.

We report a pediatric case of a 9-year-old girl who developed acute methemoglobinemia following ingestion of a cockroach gel containing thiamethoxam. She presented with nausea, vomiting, lethargy, and irritability. In the emergency department, she was in respiratory distress with hypoxia unresponsive to high-flow oxygen. Notably, her venous blood appeared dull brown. Capillary blood gas analysis revealed an elevated MetHb level of 42.1%, low pO₂, and metabolic acidosis. Intravenous methylene blue was administered promptly, resulting in rapid clinical improvement and a reduction in MetHb to 2.9% within one hour.

This case highlights a novel manifestation of thiamethoxam toxicity: life-threatening methemoglobinemia. According to current toxicological studies, thiamethoxam, a neonicotinoid primarily associated with neuromuscular and liver toxicity, has not previously been linked to hematologic effects. A plausible mechanism may involve oxidative stress from thiamethoxam metabolites, leading to hemoglobin oxidation. Timely diagnosis was made possible by high clinical suspicion and immediate blood gas analysis. The patient recovered fully with methylene blue treatment.

This case expands the known toxicologic profile of thiamethoxam and represents the first reported instance of thiamethoxam-induced methemoglobinemia. Early recognition and treatment are critical. In cases of unexplained hypoxia, especially in children with potential exposure to oxidizing agents like insecticides, clinicians should maintain a high index of suspicion for methemoglobinemia, even if the product appears benign.

## Introduction

Methemoglobinemia is a life-threatening toxicological condition characterized by the oxidation of ferrous iron (Fe²⁺) in hemoglobin to ferric iron (Fe³⁺), resulting in the formation of methemoglobin (MetHb), which is incapable of binding oxygen [1]. This leads to impaired oxygen delivery to tissues, despite a normal arterial oxygen partial pressure [2]. Clinical presentation depends on the MetHb level and may range from mild cyanosis to profound hypoxia, seizures, coma, or death [2,3]. While congenital methemoglobinemia is very rare and usually occur due to cytochrome b5 reductase deficiency, acquired forms are more common and usually result from exposure to oxidizing agents such as nitrates, sulfonamides, aniline dyes, and certain insecticides [4].

Neonicotinoids are a newer class of insecticides that act as agonists at insect nicotinic acetylcholine receptors. One such agent, thiamethoxam, is widely used due to its potent insecticidal action and presumed low toxicity in humans [5]. It is frequently marketed as a household gel, often lacking robust child-resistant packaging. Human exposures are increasing, especially in developing countries, raising concerns about underrecognized toxic effects. Reported toxicities from thiamethoxam in humans have primarily been gastrointestinal or neurologic, with minimal documented association with methemoglobinemia [6,7].

To our knowledge, this is the first pediatric case report of acute methemoglobinemia following ingestion of a thiamethoxam-containing insecticidal gel. The child presented unexplained hypoxia unresponsive to supplemental oxygen. Methemoglobinemia was diagnosed through capillary blood gas analysis, and prompt administration of intravenous methylene blue led to rapid and complete clinical recovery, with no rebound observed. This case expands the known toxicological profile of thiamethoxam and underscores the importance of considering methemoglobinemia in children presenting with cyanosis, refractory hypoxia despite oxygen therapy, or characteristically dark blood, particularly in the context of toxic ingestion. It also highlights the need for poison control reporting and increased public awareness regarding the potential dangers of household insecticides, especially in vulnerable pediatric populations.

## Case presentation

A 9-year-old girl with suspected developmental delay was brought to the emergency department approximately four hours after unwitnessed ingestion of a domestic cockroach-killing gel containing 0.05% thiamethoxam, a neonicotinoid insecticide. Following ingestion, she developed recurrent vomiting, progressive drowsiness, irritability, and decreased responsiveness. There were no reported seizures, abnormal movements, hypersalivation, or dyspnea.

On presentation, she was conscious but agitated, tachypneic, and hypoxemic. Despite oxygen therapy via a non-rebreather mask (NRB) at 15 L/min, her oxygen saturation remained in the high 80s. Physical examination revealed clear lung sounds, a soft abdomen, and unremarkable cardiovascular and neurological findings. The discrepancy between persistent hypoxia and an otherwise normal physical exam raised suspicion for a hemoglobinopathy or toxic ingestion. Table 1 presents vital signs of the patient during the hospital stay, showing changes before and after treatment.

**Table 1:** Vital signs during hospital course Vital signs recorded during the hospital stay showed initial tachycardia, elevated respiratory rate, and persistent hypoxia despite oxygen support, followed by clinical stabilization. bpm: breaths per minute, rpm: respiration per minute, NRB: non-rebreather mask.

Vitals	At presentation	At one hour	At two hours	Ten minutes after treatment	Two hours after treatment	Reference range
Temp (°C)	36.8	36.6	36.7	36.5	37.1	36.5-37.5
Pulse (bpm)	135	155	128	74	95	70-120
Respiratory rate (rpm)	26	24	22	20	19	18-30
BP (mmHg)	99/60	96/60	99/70	101/67	108/81	Age-appropriate
SpO₂ (%)	86	85	87	95	99	≥95
O₂ device	NRB	NRB	NRB	Room air	Room air	-

As there was no improvement despite supplemental oxygen, venous access was established, and chocolate-brown blood was noted, classic for methemoglobinemia. A capillary blood gas analysis revealed an elevated MetHb of 42.1%, a low pO₂ of 15.2 mmHg, and an elevated lactate of 3.5 mmol/L, all consistent with functional hypoxia. Following confirmation of a normal G6PD level, since G6PD deficiency is a contraindication to methylene blue, treatment with intravenous methylene blue was initiated. Post-treatment, serial blood gas measurements and MetHb levels demonstrated marked improvement, as shown in Table 2.

**Table 2:** Capillary blood gas and methemoglobin trend Serial capillary blood gas results and MetHb levels demonstrated rapid biochemical improvement and resolution of methemoglobinemia following methylene blue administration.

Parameters	At presentation	At one hour	At two hours	Ten minutes after treatment	Two hours after treatment	Reference range
pH	7.261	7.378	7.341	7.315	7.337	7.35-7.45
pCO₂ (mmHg)	57.1	49	43.1	48.6	43.7	35-45
pO₂ (mmHg)	15.2	207	150	53.6	85	83-108
Lactate (mmol/L)	3.5	4.6	4.0	1.1	1.0	0.5-1.6
MetHb (%)	42.1	39.6	39	2.9	0.5	0.5-1.5

The patient received intravenous methylene blue at a dose of 2 mg/kg and showed rapid clinical improvement. Her respiratory distress resolved, and oxygen saturation rose to above 94% in room air. Laboratory tests performed one hour after treatment showed a reduction in MetHb to 2.9%. She remained stable in the high dependency unit over the next 24 hours, with no rebound in MetHb levels. The patient was stable and discharged home after a 24-hour observation. Caregivers were counseled on the safe storage of household chemicals and injury prevention, particularly for children with developmental vulnerabilities.

## Discussion

Methemoglobinemia is a potentially fatal disorder in which the ferrous ion (Fe²⁺) in hemoglobin is oxidized to the ferric (Fe³⁺) state, resulting in the formation of MetHb [1]. Unlike oxyhemoglobin, MetHb cannot bind or transport oxygen, resulting in impaired oxygen delivery to tissues, even when pO₂ in the blood is normal or elevated [2]. This results in a paradoxical condition known as functional anemia, where oxygen is present but not available to tissues.

The body’s primary defense against MetHb accumulation is the cytochrome b5 reductase enzyme (also known as NADH-dependent methemoglobin reductase), which maintains MetHb levels below 1%-2% under normal conditions. However, when oxidative stress exceeds the capacity of this enzymatic system, particularly in infants or individuals with enzyme deficiencies, MetHb levels may rise significantly [3,4]. Common oxidizing agents include any local anesthetics (e.g., benzocaine, lidocaine), nitrites, nitrates, sulfonamides, aniline dyes, and pesticides [8]. In children, acquired methemoglobinemia usually results from accidental ingestion of such substances, especially in settings where pesticide labeling and storage practices are inadequate.

In our case, the suspected agent was thiamethoxam, a neonicotinoid insecticide. This class of compounds is derived from nicotine and acts as a selective agonist of insect nicotinic acetylcholine receptors, initially causing neuronal overstimulation followed by paralysis [5]. Thiamethoxam is considered to have low mammalian toxicity, primarily affecting the gastrointestinal and neuromuscular systems rather than the hematological system in cases of acute exposure. To our knowledge, this is the first reported pediatric case of methemoglobinemia presenting as the sole manifestation of thiamethoxam ingestion.

The exact mechanism by which thiamethoxam induces methemoglobinemia remains unclear. However, it is known to produce metabolites such as clothianidin, which can generate reactive oxygen species (ROS). These ROS have the potential to oxidize hemoglobin, leading to MetHb formation [9]. Toxicological studies on thiamethoxam have documented hepatotoxicity, mitochondrial dysfunction, and oxidative stress in rodent models; however, hematological parameters were often omitted or not thoroughly assessed in these investigations [10,11]. As a result, hematologic adverse effects may be underrecognized or underreported in both clinical settings and toxicovigilance databases.

From a clinical perspective, methemoglobinemia should be considered in patients presenting with hypoxia that does not improve with oxygen administration, especially when there is a history of ingestion or exposure, or when chocolate-brown blood is observed. Pulse oximetry readings in such cases can be misleading, often plateauing around 85% despite high-flow oxygen administration [12]. Capillary or venous blood gas analysis with co-oximetry remains the gold standard for diagnosis, allowing direct measurement of MetHb concentration [13]. In our case, the child's oxygen saturation remained in the high 80s despite being on NRB. Diagnosis was swiftly confirmed through capillary blood gas analysis, which showed MetHb levels exceeding 42%.

The treatment of choice for symptomatic or severe methemoglobinemia is intravenous methylene blue, a synthetic dye that serves as an alternative electron donor in the NADPH-dependent MetHb reductase pathway [14,15]. A single dose of 1-2 mg/kg typically results in rapid improvement, as was observed in our patient. It is important to note that methylene blue is contraindicated in individuals with glucose-6-phosphate dehydrogenase (G6PD) deficiency due to the risk of hemolysis [14,15]. Our patient tested negative for G6PD deficiency and responded promptly without complications or rebound.

An additional noteworthy aspect of this case is the potential role of neurodevelopmental vulnerability in the accidental ingestion. Children with autism spectrum disorder or developmental delays are at increased risk of exploratory behaviors, which may lead to ingestion of non-food substances, including hazardous household chemicals [16]. This underscores the importance of preventive measures such as child-resistant packaging, proper labeling, and caregiver education, particularly in settings with limited access to healthcare.

This case also emphasizes the critical role of poison center consultation and toxicovigilance reporting. Safety authorities rely on case reports, both published and anecdotal, to update toxicologic profiles, set exposure thresholds, and recognize rare side effects. Currently, neither the National Poisons Information Service (NPIS) nor the Environmental Protection Agency (EPA) lists methemoglobinemia as a known hematologic risk of thiamethoxam exposure [7,17]. This case not only expands the known toxicological spectrum of thiamethoxam but may also contribute to future updates of clinical guidelines and safety databases.

## Conclusions

This case highlights an atypical and potentially life-threatening presentation of acquired methemoglobinemia following thiamethoxam ingestion in a pediatric patient. Although neonicotinoids, such as thiamethoxam, are generally considered to have low toxicity in humans, this case report challenges that assumption by demonstrating a hematologic effect previously unreported in pediatric toxicology literature.

A high index of clinical suspicion, prompted by signs such as refractory hypoxia and chocolate-brown blood, enabled early diagnosis and timely administration of methylene blue, resulting in rapid and sustained recovery. This case underscores the need for increased toxicovigilance, heightened awareness of rare pesticide-related adverse effects, and early intervention, particularly in vulnerable populations such as children with developmental disabilities.
